# Entropy Production and the Pressure–Volume Curve of the Lung

**DOI:** 10.3389/fphys.2016.00073

**Published:** 2016-03-01

**Authors:** Cláudio L. N. Oliveira, Ascânio D. Araújo, Jason H. T. Bates, José S. Andrade, Béla Suki

**Affiliations:** ^1^Departamento de Física, Universidade Federal do CearáFortaleza, Brazil; ^2^Department of Medicine, University of VermontBurlington, VT, USA; ^3^Department of Biomedical Engineering, Boston UniversityBoston, MA, USA

**Keywords:** entropy production, lungs modeling, fibrosis, emphysema, thermodynamic modeling

## Abstract

We investigate analytically the production of entropy during a breathing cycle in healthy and diseased lungs. First, we calculate entropy production in healthy lungs by applying the laws of thermodynamics to the well-known transpulmonary pressure–volume (*P*–*V*) curves of the lung under the assumption that lung tissue behaves as an entropic spring similar to rubber. The bulk modulus, *B*, of the lung is also derived from these calculations. Second, we extend this approach to elastic recoil disorders of the lung such as occur in pulmonary fibrosis and emphysema. These diseases are characterized by particular alterations in the *P*–*V* relationship. For example, in fibrotic lungs *B* increases monotonically with disease progression, while in emphysema the opposite occurs. These diseases can thus be mimicked simply by making appropriate adjustments to the parameters of the *P*–*V* curve. Using Clausius's formalism, we show that entropy production, Δ*S*, is related to the hysteresis area, Δ*A*, enclosed by the *P*–*V* curve during a breathing cycle, namely, Δ*S*=Δ*A*∕*T*, where *T* is the body temperature. Although Δ*A* is highly dependent on the disease, such formula applies to healthy as well as diseased lungs, regardless of the disease stage. Finally, we use an *ansatz* to predict analytically the entropy produced by the fibrotic and emphysematous lungs.

## 1. Introduction

The laws of thermodynamics are based on empirical evidence derived from the behavior of macroscopic systems (Fermi, 1956), and in this respect share similarities with much of our knowledge about biological systems. Indeed, in his seminal 1944 book “What is life?,” Erwin Schrödinger addressed the question of how living systems can maintain order in apparent violation of the second law of thermodynamics. He postulated that life is only possible if living systems export entropy to their surroundings (Schrödinger, 1944). He also conjectured the existence of an “aperiodic crystal” containing the genetic information of living beings a decade earlier than the discovery of DNA. His influential ideas stimulated the development of molecular biology and many areas of theoretical biology that are still being pursued today (Dyson, 1999).

The field of thermodynamics has been greatly advanced by the advent of the digital computer which provides the means to link thermodynamics to microscopic mechanisms using the ideas of statistical mechanics in situations that defy analytical calculation. This is also now finding significant application in biology. For example, the microscopic progression of fibrosis and emphysema in the lung has been linked to pathological changes in macroscopic lung function in terms of a percolation process (Bates et al., 2007; Oliveira et al., 2014) and the fractal dimension of nuclear chromatin has been found to provide a potential molecular tool for cancer prognosis (Metze, 2013). Additionally, the connectivity of the brain has been studied in the framework of complex networks (Reis et al., 2014) as well as the maximization of entropy production (Seely et al., 2014). These advances often rely on extensive numerical computation because of the highly non-linear interactions involved between the myriad components in these complex systems.

Regardless of these complexities, however, the laws of thermodynamics must still hold. This applies in particular to the second law that governs entropy. The very essence of a living system is continual internal activity of a very ordered nature, but this activity necessarily generates entropy which is the engine of disorder. Nevertheless, living systems manage to maintain, throughout their lifetimes, all electrical, chemical, and temperature gradients that define their internal order (Annamalai and Silva, 2012). Accordingly, living systems must somehow export the entropy they generate to the environment, as Schrödinger postulated (Schrödinger, 1944). But what happens if not all the entropy is exported? The remaining entropy stays within the system where its inescapable consequence must be a gradual progression of the system toward malfunction (i.e., disease) and eventual death. This raises two considerations that are paramount for the life and health of an organism: (1) the rate at which entropy is produced, and (2) the success with which that entropy is exported. In this paper we focus on the first of these considerations in relation to the lung, a well-defined thermodynamic system in the human body that exchanges mass and energy continually with its surroundings.

We first derive a relation describing entropy production in the lung and apply it to two pulmonary diseases that affect the elastic protein fibers of the lung tissue, of which there are two main kinds. *Pulmonary fibrosis* involves the excess production and abnormal arrangement of protein fibers and thus causes the lung to become stiffer than normal, while *emphysema* involves the destruction of these fibers and so leads to a lung that is correspondingly less stiff than normal (Levitzky, 1995). Currently, the role thermodynamics plays in these diseases of the extracellular matrix is not well-understood. Accordingly, in the present study we propose a simple thermodynamic model of the pressure–volume (*P*–*V*) relationship of the lung. We use this model to calculate the entropy produced in the lung during normal breathing, and then examine how this production is altered in pulmonary fibrosis and emphysema.

## 2. Thermodynamics of healthy lungs

The volume of fresh air inspired with every breath is a consequence of the pressure generated by the respiratory muscles (principally the diaphragm) and the elasticity of the lung tissues. The latter include contributions from both the protein fibers of the extracellular matrix and the surface tension of the air–liquid interface (Suki et al., 2011b). These events take place under essentially isothermal conditions because temperature fluctuations deep in the lung are negligible even though the temperature of the inspired air gradually increases from ambient at the mouth to body temperature at some point along the conducting airway tree (McFadden etal., 1985). A thermodynamic model has already been developed to predict the work done on the air–liquid interface in the lung as a result of surface tension (Prokop et al., 1999), something that can change markedly in, for example, acute respiratory distress syndrome (Gregory et al., 1991).

**Figure 1:**
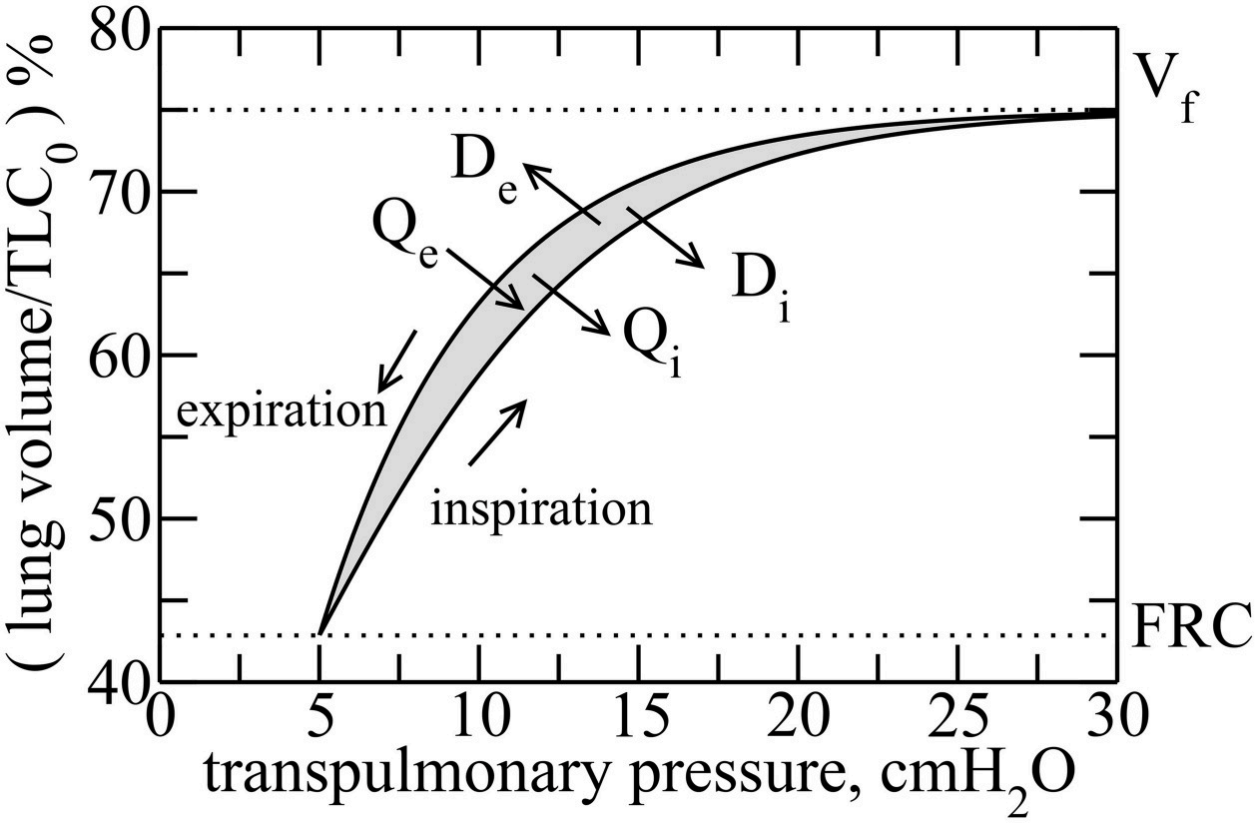
**Typical transpulmonary pressure vs. volume curves in healthy lungs**. By considering the rubber approach, reversible heat *Q*_*i*_ is released during inflation, whereas it is absorbed during deflation *Q*_*e*_. However, due to dissipation, heat is released during both inspiration and expiration, denoted by *D*_*i*_ and *D*_*e*_, respectively. The hysteresis is due to an asymmetry between the recruitment and derecruitment processes of collagen fibers, during inspiration and expiration, respectively. The hysteresis area, Δ*A*, is highlighted in gray.

We consider here the lung as a system with a state defined by its volume (*V*). The equilibrium state is the value of *V* at the end of a relaxed expiration, known as Functional Residual Capacity (*FRC*), which is also taken here as the minimum *V*. During inspiration, a pressure gradient across the lung, known as transpulmonary pressure (*P*), expands the lung to a volume *V*_*f*_ that is typically somewhat variable from breath to breath during normal respiration but which has a maximum voluntary value known as Total Lung Capacity (*TLC*). During expiration, *V* is returned to functional residual capacity (*FRC*) by the elastic recoil forces generated within the lung tissues during the previous inspiration. Figure 1 shows typical *P* vs. *V* (*P*–*V*) curves for the lung. Such curves are well-known and can be measured experimentally (Venegas et al., 1998). Note that *V* here represents the volume of air entering and leaving the lung during breathing, not the volume of the lung tissue.

The elastic recoil pressure of the lungs is generated as a result of microscopic processes occurring within the lung tissue, such as the stretching and unfolding of individual protein fibers. We assume here that the lung tissue behaves similarly to rubber which is an elastic material composed of long-chain polymers, called elastomers, that have particular thermodynamic properties. For example, the Young's modulus of rubber is proportional to absolute temperature, an intriguing property that causes rubber to release heat when stretched as a result of a corresponding decrease in entropy, and conversely to absorb heat when returning toward equilibrium (Callen, 1985). Microscopically, the decrease in entropy can be explained by progressively fewer molecular conformations available for the elastomers as they stretch. Conversely, the decreased entropy in the stretched state gives rubber the ability to subsequently convert thermal energy into work as it contracts against a load and its entropy increases. In this sense, a rubber behaves somewhat like an ideal monatomic gas because neither stores potential energy in the distortion of chemical bonds, but both convert thermal energy into work on their surroundings (Brown, 1963).

The justification for considering lung elasticity to have an entropic basis comes first of all from the fact that the principal structural proteins in lung tissue are elastin and collagen, both of which are organized into long tortuous fibers. For both elastin Baldock et al. (2011) and collagen Buehler and Wong (2007) these fibers have been modeled, for modest stretches, as worm-like chains that behave like entropic springs (although at high levels of strain both fiber types begin to store elastic energy in their molecular bonds). Collagen is at least 100 times stiffer than elastin, so for simplicity we will assume that collagen fibers are actually infinitely stiff so that worm-like chain entropy applies only to the elastic fibers. Entropy also applies to collagen fibers but for a different reason, as follows. The collagen and elastic fibers form an essentially random network in which the stress at low strain is borne almost exclusively by the elastin fibers and the collagen fibers are flaccid and wavy. Notice that we are simplifying the collagen network effects by assuming that the collagen fibers are not extensible and that the major contributor to the stress is the recruitment of the stiff collagen by folding as was done previously (Maksym and Bates, 1997). As lung volume increases the regional tissue stress also increases, and the collagen fibers become taught and thus prevent those elastin fibers in their immediately vicinity from being able to stretch. This gives rise to a progressive stiffening of the entire tissue as *V* increases, as seen in the *P*–*V* curve (Figure 1). However, the collagen fibers in 3D lung tissue are not entirely constrained in their orientations but rather may assume different directions as a result of thermal motion (Bates, 1998). At equilibrium these fiber directions may be quite random but as the tissue stretches the fibers become oriented preferentially in the direction of local strain. This reduces the number of possible configurations of the fibers within the tissue matrix and hence reduces their entropy. Assuming that the fibers resist being oriented in the direction of strain to a degree that is proportional to absolute temperature, *T*, collagen recruitment can also be modeled as an entropic process similar to the stretching of rubber.

We can thus reason that the collagen and elastin fibers in lung tissue ought to behave together as an entropically elastic material. Note, however, that these fibers do not undergo their thermodynamic excursions within the living lung in isolation but rather exist under essentially isothermal conditions because the metabolic processes of life, and especially the heat-exchanging capacity of the circulating blood, maintain core body temperature at an even 37°C. Consequently, these fibers have the capacity to exchange heat with their environment and thus to dissipate energy, which occurs as a consequence of the frictional heat that is generated as the fibers are continually jostled by thermal motion. Thus, an amount of heat energy *D*_*i*_ is released irreversibly to the surroundings as a result of frictional losses during inspiration. Similarly, during expiration an amount *D*_*e*_ is released irreversibly as frictional losses. Note that these frictional heats are different to the heats released during inspiration and imported during expiration as a result of entropic changes, namely *Q*_*i*_ and *Q*_*e*_, respectively. In other words, even though the macro-configurations of the collagen and elastin fiber systems may be identical at the end of each expiration, their micro-configurations are different from breath-to-breath, and frictional energy is dissipated in moving from one end-expiratory micro-configuration to the next.

Clausius formulated the Second Law as follows:

(1) $$\begin{array}{l} N=S-S_{0}-\int \frac{dQ}{T}, \end{array}$$

where *N* > 0 is the so-called uncompensated transformation, which is the entropy due to irreversible processes within the system. *S* and *S*_0_ are the entropies of the final and initial states, respectively, and *T* is the absolute temperature. The last term identifies any exchange of heat with the environment. Hence, Δ*S*_*i*_ = *S*−*S*_0_ represents the entropy production during an irreversible process that moves the system from the initial to the final state. In our case, since the lung returns at the end of each breath to the same volume, *FRC*, at the same temperature, *T*, the entropy of the tissues at the end of a breath cycle should be the same as at the end of the previous cycle. This implies that the entropy produced by the irreversible processes is exported to the environment, principally the heat bath provided by the circulation.

Now, the change in entropy Δ*S*_*r*_ around the cycle due to the alterations in the configurations of the elastin and collagen fibers must be zero because we consider the elastic properties of lung tissue to be conservative. In other words, the last term in Equation (1) cancels during the cycle:

$$\begin{array}{l} \Delta S_{r}=\frac{-Q_{i}+Q_{e}}{T}=0, \end{array}$$

which also means that the change in entropy of the system is entirely due to the frictional work, *N* = Δ*S*_*i*_, which is given by

(2) $$\begin{array}{l} \Delta S_{i}=\frac{D_{i}+D_{e}}{T}>0, \end{array}$$

where *D*_*i*_ and *D*_*e*_ are the amounts of frictional energy dissipated during inspiration and expiration, respectively.

On the other hand, the sum of *D*_*i*_ and *D*_*e*_ is the total frictional energy dissipated around the breath cycle, which equals the hysteresis area of the *P*–*V* loop (Figure 1). This area, shown in gray in the figure, is

(3) $$\begin{array}{l} \Delta A=\int_{FRC}^{V_{f}}P_{i}dV+\int_{V_{f}}^{FRC}P_{e}dV=D_{i}+D_{e}, \end{array}$$

where *P*_*i*_ and *P*_*e*_ are simply *P* during inspiration and expiration, respectively. Substituting into Equation (2) then gives

(4) $$\begin{array}{l} \Delta S_{i}=\frac{\Delta A}{T}, \end{array}$$

where Δ*S*_*i*_ is positive since Δ*A* is positive. Notice that if the area between the curves vanishes, *N* in Equation (1) also vanishes as predicted by the Clausius formulation for reversible processes. Equation (4) shows that the energy dissipated during each breathing cycle can be linked directly to entropy production, Δ*S*_*i*_, which is exported to the environment with each breath.

## 3. Analytical fittings of the transpulmonary *P*–*V* curves

We can obtain a formula for Δ*S*_*i*_ from analytical expressions for the inspiratory and expiratory *P*–*V* curves shown in Figure 1. These curves can be fitted with sigmoidal and exponential functions, respectively, as follows (Venegas et al., 1998),

(5) $$\begin{array}{rcl} V_{i} & = & \frac{V_{f}}{1+e^{-\left(\frac{P-P_{FRC}-a}{b}\right)}},and\\ V_{e} & = & V_{f}-\Delta Ve^{-\left(\frac{P-P_{FRC}}{k}\right)}, \end{array}$$

where *V*_*i*_ and *V*_*e*_ represent *V* during inspiration and expiration, respectively. The difference Δ*V* = *V*_*f*_−*FRC* is the change in lung volume during a breath, and is usually referred to as tidal volume. Note that *V*_*f*_ is substantially smaller than *TLC* during normal resting breathing. During inspiration, *V*_*i*_ begins at its minimum value of *FRC* (when *P* = *P*_*FRC*_) and increases to *V*_*f*_, in which case the parameter *a* = *b*ln(Δ*V*∕*FRC*) represents the inflection point of the sigmoid. *P*_*FRC*_ is a constant representing the pressure at the lowest lung volume *FRC*. The parameter *b* governs the slope of the sigmoid at its inflection point; the larger is *b* the smaller is the slope. The exponential equation for *V*_*e*_ in Equation (5) is governed by the exponent *k* that, like *b*, determines the rate of change of volume with pressure except this time during expiration. Notice that, these functions for *V*_*i*_ and *V*_*e*_ meet each other at *FRC* and *TLC*.

Rewriting Equation (5) explicitly in terms of *P* gives

(6) $$\begin{array}{rcl} P_{i} & = & P_{FRC}+a+b\ln\;\left(\frac{V}{V_{f}-V}\right),and\\ P_{e} & = & P_{FRC}+k\ln\;\left(\frac{\Delta V}{V_{f}-V}\right). \end{array}$$

Finally, integrating these equations with respect to *V* and substituting into Equation (3) gives

(7) $$\begin{array}{l} \Delta S_{i}=\frac{1}{T}\left[bV_{f}\ln\left(\frac{V_{f}}{FRC}\right)-k\Delta V\right]. \end{array}$$

This equation defines the entropy produced (and exported) by the lung tissue during a single breathing cycle as a function of the tidal volume, *V*_*f*_. The parameters *b*, *k*, *FRC* and *T* can be taken to be constants for a normal adult lung, but may vary with disease.

## 4. Bulk modulus

The bulk modulus of the lung is the inverse of its specific compliance and characterizes its elastic properties; the larger the bulk modulus, the stiffer (less compliant) the lung. The bulk modulus *B* is thus defined as

$$\begin{array}{l} B=V\frac{dP}{dV}. \end{array}$$

Using Equation (6) one finds that *B* during inspiration and expiration is given by,

$$\begin{array}{rcl} B_{i} & = & b\frac{V_{f}}{V_{f}-V},and\\ B_{e} & = & k\frac{V}{V_{f}-V}, \end{array}$$

respectively. Because of the non-linear *P*–*V* relationships, *B* changes with *V* during both inspiration and expiration. For simplicity, therefore, we will consider a representative *B* at the halfway point of the breath, i.e., at *V* = *V*_*f*_∕2, which gives *B*_*i*_ = 2*b* and *B*_*e*_ = *k*. Moreover, it is always observed experimentally that *B*_*i*_ > *B*_*e*_, so in the following we will use *k* = *b*, which satisfies this condition.

## 5. Applying the model to fibrotic and emphysematous lungs

It has been observed that Equation (6) also provide good fits to the *P*–*V* curve of both fibrotic (Ferreira et al., 2011) and emphysematous (Soutiere and Mitzner, 2004; Pérez-Rial et al., 2014) lungs. The altered *P*–*V* curves in these diseases can thus be mimicked simply by adjusting the parameters in Equation (6). In fibrosis the lung becomes stiffer so patients need to apply more pressure to inspire a smaller volume of air. In emphysema the loss of lung elasticity increases *FRC* due to the outward recoil of the chest wall.

**Figure 2:**
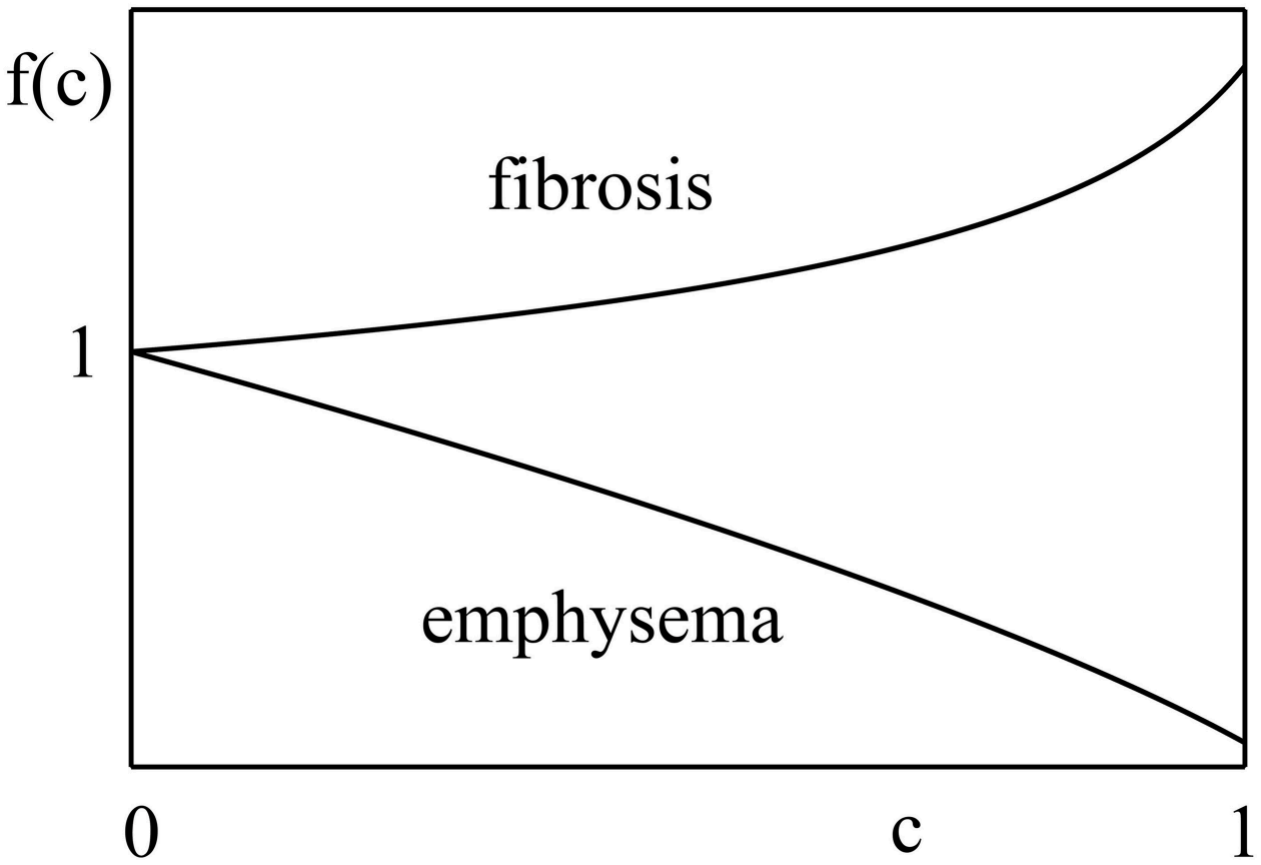
**Function ***f***(***c***) behaves differently for different diseases**. In fibrosis, it increases with fraction of lung parenchymal tissue affected by disease, *c*, while, in emphysema, it decreases with the disease stage. Although, the explicit nature of *f*(*c*) is unknown, it should start from unity and change monotonically with *c*.

Following the results observed in Ferreira et al. (2011), Soutiere and Mitzner (2004), and Pérez-Rial et al. (2014), we model fibrosis by increasing *b*, while emphysema is modeled by decreasing *b*. Specifically, we let *b* vary with disease state according to

$$\begin{array}{l} b=k=b_{0}f\left(c\right), \end{array}$$

where *c* is the fraction of lung parenchymal tissue affected by disease (a measure of disease severity), *b*_0_ is the value of *b* for a healthy lung, and *f*(*c*) is a dimensionless function capturing the influence of the disease. It starts from 1, at *c* = 0, and increases (decreases) monotonically with *c* for fibrosis (emphysema). Thus, *b* starts at *b*_0_ and changes monotonically either up or down as the disease progresses. Figure 2 shows schematically how *f*(*c*) changes for fibrosis and emphysema.

Another important physiological change that occurs in both fibrosis and emphysema is that *V*_*f*_ also changes with disease progression, so *V*_*f*_ is also a function of *c*. Specifically, *V*_*f*_(*c*) decreases in fibrosis and increases in emphysema. This has the effect of essentially creating a smaller or larger lung, respectively, which means that the ratio of *V*_*f*_ to *FRC* in Equation (7) remains unchanged. *V*_*f*_ and *FRC* thus change in the same proportion according to a function *g*(*c*) as follows:

$$\begin{array}{rcl} FRC\left(c\right) & = & FRC_{0}g\left(c\right),and\\ V_{f}\left(c\right) & = & V_{f0}g\left(c\right), \end{array}$$

where *FRC*_0_ and *V*_*f*0_ are the healthy values for *FRC* and *V*_*f*_, respectively. Like *f*(*c*), *g*(*c*) also represents the influence of the disease and also starts from 1, at *c* = 0, and changes monotonically with *c* but in the opposite direction. That is, *f*(*c*) increases in fibrosis while *g*(*c*) decreases to account for the fractional change in lung volume that occurs with disease progression. Conversely, *f*(*c*) decreases in emphysema while *g*(*c*) increases.

We are now in a position to describe how the entropy production per breathing cycle changes as disease evolves. Consider, for example, the case of a deep inspiration to *TLC* (i.e., *V*_*f*_ = *TLC*_0_). We can then compare the behavior of *P*–*V* curves in diseased lungs to healthy lungs. This gives, from Equation (7),

(8) $$\begin{array}{l} \Delta S_{i}\left(c\right)=\frac{b_{0}f\left(c\right)g\left(c\right)}{T}\left[TLC_{0}\ln\;\left(\frac{TLC_{0}}{FRC_{0}}\right)-\Delta V_{0}\right], \end{array}$$

where Δ*V*_0_ = *TLC*_0_−*FRC*_0_.

Equation (8) shows that the entropy produced in the lungs over the breath cycle changes with disease by an amount given simply by multiplying Δ*S*_*i*_ (from Equation 7) by the product of *f*(*c*) and *g*(*c*). This shows how the alteration of bulk modulus, as well as the alteration in the parameter *b* in disease, plays a role in entropy production. Additionally, one interpretation of entropy production is that its increase in a given disease condition signifies a less efficient mechanical function for the lung and more of the elastic recoil is converted into heat.

## 6. Ansatz for *f*(*c*) and *g*(*c*)

It remains to define *f*(*c*) and *g*(*c*) for either fibrosis or emphysema. Conceivably, these functions could be determined by analyzing *P*–*V* curves at different stages of the disease, but this has yet to be done. Alternatively, the functions could be guessed at on the basis of the behavior of a computational model of disease progression, such as the percolation model we have previously investigated (Oliveira et al., 2014). To keep things simple at this point, however, we take here an empirical approach by first noting that *f*(*c*) and *g*(*c*) should start at unity and change monotonically with the progression of disease. Furthermore, it is known that the symptoms of fibrosis only become apparent when about 30% of the lung is affected, while emphysema symptoms may be noticed at an earlier stage. In other words, the function *f*(*c*) for fibrosis should not change much until *c* ≈ 0.3, whereas in emphysema symptoms may occur for *c* ≳ 0.

Accordingly, we make the following assumptions for the *f* functions for fibrosis, *f*_*fi*_(*c*), and for emphysema, *f*_*em*_(*c*):

$$\begin{array}{rcl} f_{fi}\left(c\right) & = & {\left(1-c\right)}^{-\alpha },and\\ f_{em}\left(c\right) & = & e^{-{\left(\frac{c}{\beta }\right)}^{2}}. \end{array}$$

The equation for *f*_*fi*_(*c*) mimics the fact that fibrosis progresses slowly at early stages but grows faster as the affected tissue nears the percolation threshold in the lung. The equation for *f*_*fi*_(*c*) and *f*_*em*_(*c*) captures the behavior of the bulk modulus of emphysematous lungs as found in previous studies (Parameswaran, 2011; Oliveira et al., 2014).

The function *g*(*c*), which defines how *TLC* and *FRC* change with the disease, is actually harder to predict without experimental data. It has been reported, however, that the *P*–*V* area and the dissipation during breathing increases both in fibrosis (Manali et al., 2011) and emphysema (Ito et al., 2004). Here, for simplicity, we apply a similar analytical approach as that used for *f*(*c*). That is,

$$\begin{array}{rcl} g_{fi}\left(c\right) & = & {\left(1-c\right)}^{\gamma },and\\ g_{em}\left(c\right) & = & e^{{\left(\frac{c}{\kappa }\right)}^{2}}, \end{array}$$

for fibrosis and emphysema, respectively. If γ and κ are positive, then *g*_*fi*_(*c*) decreases while *g*_*em*_(*c*) increases with *c*. In addition, in order for Δ*A* to increase with *c* for both diseases as reported in the literature (Ito et al., 2004; Manali et al., 2011) the following conditions must be met: α > γ and β > κ.

**Figure 3:**
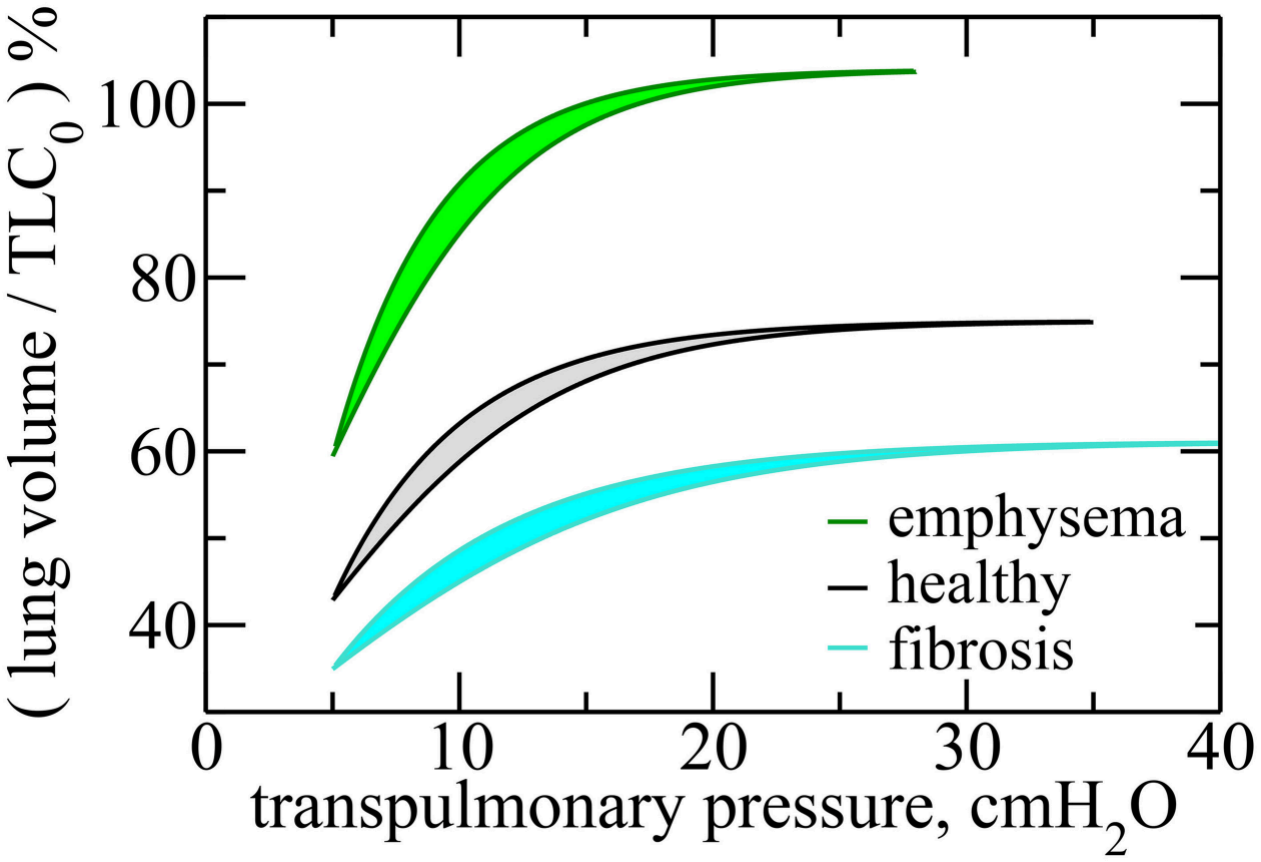
**Transpulmonary ***P***–***V*** curves for healthy, fibrotic, and emphysematous lungs, for ***c*** = 0.4**. For fibrosis, the curves are obtained for $f_{fi}\left(c\right)={\left(1-c\right)}^{-\alpha }$, where α = 0.6, and $g_{fi}\left(c\right)={\left(1-c\right)}^{\gamma }$, where γ = 0.4. For emphysema, $f_{em}\left(c\right)=e^{-{\left(\frac{c}{\beta }\right)}^{2}}$, where β = 0.9, and $g_{em}\left(c\right)=e^{{\left(\frac{c}{\kappa }\right)}^{2}}$, with κ = 0.7. For these curves we use the following parameters, *b*_0_ = 5.0*cmH*2*O*, *FRC*_0_ = 3.0*l*, and *P*_*FRC*_ = 5.0*cmH*2*O*. Notice that the lung volume is calculated in terms of *TLC*_0_, the Total Lung Capacity for healthy lungs, given by *TLC*_0_ = 7.0*l*. Here, *V*_*f*_ was taken to be 75% of *TLC* in order to represent a typical volume at end-inspiration. The hysteresis areas, for each of these cases, are highlighted in different colors.

**Figure 4:**
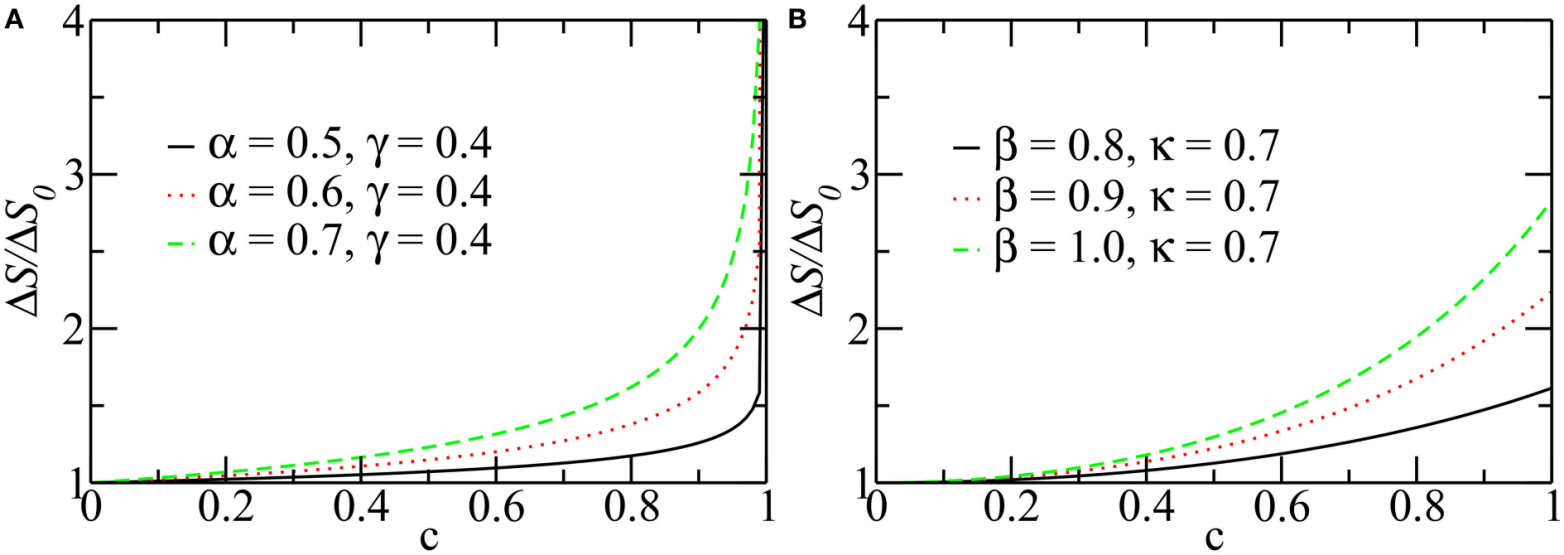
**The entropy production per breathing cycle as a function of ***c*****. For different values of parameters, α, β, γ, and κ, in fibrosis **(A)** and emphysema **(B)**. Notice that the entropy production is normalized with its value at *c* = 0. For this calculation *V*_*f*_ was taken equal to *TLC*, so the curves represent the maximum production of entropy during a breathing. The other parameters are the same as those used in Figure 3.

Figure 3 shows the *P*–*V* curves plotted using these analytical expressions for healthy lungs, as well as fibrotic and emphysematous lungs, for several sets of parameters (see the caption of the figure). These curves start at *P* = *P*_*FRC*_, the pressure at *FRC* corresponding to one of the lung conditions (fibrosis, emphysema, or healthy lung) and the disease severity characterized by *c*. For healthy lungs (black curves in Figure 3), we used a reference value of *FRC* of 3.0*l*. For fibrotic and emphysematous lungs (blue and green curves in Figure 3), the value of *FRC* is computed according to the function *g*(*c*) defined above, for *c* = 0.4, γ = 0.4, and κ = 0.7, giving, respectively, 2.45 and 4.17*l*. These values of *FRC*, 3.0, 2.45, and 4.17*l*, correspond, respectively, to 43%, 35%, and 60% of the *TLC*_0_ of the healthy lungs. The hysteresis area, Δ*A*, between inflation and deflation curves, for each disease and the healthy lung is colored differently. Figure 4 shows the entropy production as a function of *c* for fibrosis (Figure 4A) and emphysema (Figure 4B). Notice the sudden increase of entropy production for *c* > 0.8 in fibrosis, which suggests that in end-stage disease respiration becomes highly inefficient as much of the elastic energy stored in the fibers by the respiratory muscles is dissipated as heat. On the other hand, in emphysema, the entropy production increases much more slowly suggesting a more gradual deterioration of the efficiency of the lung.

## 7. Discussion

Entropy is a corner stone of thermodynamics and statistical mechanics and its formulation is largely independent of the microscopic details of the system, which is the basis for its wide generality and applicability. In order to understand how open systems, including all biological entities, function under far from thermodynamic equilibrium conditions, it is necessary to consider entropy production. In this study we derived a simple relation between entropy production and the hysteresis loop of the *P*–*V* curve of the lung in terms of lung volumes and parameters of lung elasticity. The model has been extended to two major diseases of the lung tissue, pulmonary fibrosis and emphysema. Currently, neither fibrosis nor emphysema can be cured, yet together they constitute an enormous public health burden; fibrosis affects approximately 5 million people worldwide (Meltzer and Noble, 2008), while the World Health Organization reports that emphysema led to the death of more than 3 million people in 2012 alone^1^ (Maksym and Bates, 1997).

Our model, however, is based on a number of assumptions and thus has important limitations. First, we neglect the contribution of surface tension at the air–liquid interface to the mechanical behavior of the lung. However, surface tension (Bachofen et al., 1970) and, more importantly, airway closure and re-opening (Gaver et al., 1985) are important issues at low lung volumes and in diseases that are accompanied by edema formation (Gregory et al., 1991). The effect of surface tension is much less in the normal lung and in emphysema and fibrosis than in acute lung injury (Massa et al., 2008). Indeed, the shape of the *P*–*V* curve reflects surfactant function to a significant extent only if the *P*–*V* curve starts from the degassed state or residual capacity where airways and alveoli are unstable and collapse since lung hysteresis above *FRC* and tissue strip hysteresis, which does not include surfactant, are nearly identical (Sakai et al., 2001). As the lung volume is inflated from these low volumes the closed lung units pop open and contribute to the hysteresis of the *P*–*V* curve (Salmon et al., 1981; Suki et al., 1994). Once lung volume exceeds *FRC*, however, stretching of elastin starts to exert a significant influence on the shape of the *P*–*V* relationship. At high lung volumes the progressive recruitment of collagen fibers becomes the dominant mechanism determining the *P*–*V* curve (see Suki et al., 2011b).

A thermodynamic formulation of the *P*–*V* curve has been derived previously by Prokop et al. (1999). However, this model only considers the effect of surface tension and neglects the contribution of tissue elasticity, and thus its validity is confined to low lung volumes. It also does not apply to lung diseases such as fibrosis and emphysema that are characterized by changes in the extracellular matrix. On the other hand, we specifically include only the contributions from tissue elasticity in our model because we restrict lung volume to being above *FRC* (Figure 1) where the contributions of surface tension to recruitment and derecruitment are presumably small. Nevertheless, our model is based on empirical curve fitting of experimental data from the literature and therefore implicitly includes contributions from surface tension reflected in these data.

We also neglect the elastic contribution of collagen to lung elastic recoil. Instead, we argue that fiber alignment and recruitment can be modeled as a change in collagen fiber configuration, an assumption that still has to be experimentally verified. We also neglect the explicit mechanisms at the microscale that likely contribute to entropy production in the tissue. For example, in several previous studies, it has been argued that polymer reptation (Suki et al., 1994), fiber alignment (Bates, 1998), fiber–fiber interactions (Mijailovich et al., 1993), and collagen–proteoglycan interactions (Suki et al., 2011a) might contribute to the dissipative processes in the lung tissue.

## 8. Conclusions

We have developed a thermodynamic model of the mechanics of breathing that gives a central role to entropic changes in the lung tissue. We applied the Clausius formalism to the transpulmonary pressure–volume (*P*–*V*) curves of a healthy lung and consider the lung tissue to behave as an entropic spring similar to rubber. Our results show that entropy production is directly related to the hysteresis area enclosed by the *P*–*V* curve during a breathing cycle. We used this model to predict how fibrosis and emphysema alter entropy production in the lung over the breathing cycle. Interestingly, our results show that both fibrotic and emphysematous lungs produce more entropy than healthy lungs. The more advanced the disease is, the more entropy is produced. This is a consequence of the hysteresis area enclosed by the *P*–*V* curves that is increased in both diseases. An interesting implication of this result is that entropy production per breathing cycle appears to be minimized in the normal lung. Because entropy production is related to energy dissipation, its minimization allows the highest efficiency for the respiratory muscles to drive breathing.

The question remains as to whether all the entropy that is produced in this manner is actually exported to the environment, or part of it is retained in the lung so that, over time, the organized structure of the lung deteriorates as a manifestation of aging and/or disease. Finally, in the diseased state, the extracellular matrix organization should be suboptimal, and hence stretching and folding of the protein network generate suboptimal entropy release. Such inefficiency may contribute to respiratory muscle fatigue and failure. Therefore, our work should stimulate new experimental studies to shed light on the relationship between incomplete release of entropy and microscopic changes in the protein network of the extracellular matrix.

## Author contributions

All authors listed, have made substantial, direct and intellectual contribution to the work, and approved it for publication.

### Conflict of interest statement

The authors declare that the research was conducted in the absence of any commercial or financial relationships that could be construed as a potential conflict of interest.

## Footnotes

^1^http://www.who.int/mediacentre/factsheets/fs315/en/ (accessed July 16, 2015)
